# CURVATURE-BASED MACHINE LEARNING METHOD FOR AUTOMATED SEGMENTATION OF DENDRITIC SPINES

**DOI:** 10.64898/2025.12.04.692360

**Published:** 2025-12-08

**Authors:** ABDEL KADER A. GERALDO, MICHAEL A. CHIRILLO, KRISTEN M. HARRIS, THOMAS G. FAI

**Affiliations:** † Department of Mathematics, Brandeis University, Waltham, MA, USA.; § Department of Biological Sciences, University of Rhode Island, Kingston, RI, USA.; ‡ Department of Neuroscience, University of Texas at Austin, Austin, TX, USA.; * Department of Mathematics and Volen Center for Complex Systems, Brandeis University, Waltham, MA, USA.

## Abstract

Recent advances in connectomics have been led by high-resolution reconstruction of large volumes of neural tissues using electron microscopy (EM), providing unprecedented insights into brain structure and function. Dendritic spines—dynamic protrusions on neuronal dendrites—play crucial roles in synaptic plasticity, influencing learning, memory, and various neurological disorders. However, current spine analysis methods often rely on manual annotation of subcellular features, limiting their ability to handle the complexity of spines in dense dendritic networks. This paper introduces a novel automated computational framework that integrates discrete differential geometry, machine learning, and 3D image processing to analyze dendritic spines in these intricate environments. By generating distributions of spine morphology from high resolution images including many thousands of spines, our approach captures subtle variations in spine shapes, offering a nuanced understanding of their roles in synaptic function. This framework is tested on multiple EM datasets, with the aim of enhancing our understanding of synaptic plasticity and its alterations in disease states. The proposed method is poised to accelerate neuroscience research by providing a scalable, objective, and comprehensive solution for spine analysis, uncovering insights into the role of spine geometry for neural function.

## 1. Introduction

Recent advances in electron microscopy and connectomics have made it possible to reconstruct astonishing large volumes of neural tissue at nanometer resolution (EM) [20, 24]. These breakthroughs have opened new opportunities for studying brain microstructures, particularly dendritic spines, dynamic protursion on dendrites where most excitatory synapses in the brain occur. The morphology of dendritic spines—encompassing shape, size, and density—is highly plastic and closely tied to fundamental neurological processes such as learning and memory [5, 14, 18, 37, 50]. Changes in spine structure have also been linked to drug abuse, environmental influences, and a wide range of neurodevelopmental, neurodegenerative, and psychiatric disorders [11, 29, 48].

Accurate detection and analysis of dendritic spines in three-dimensional reconstructions is therefore essential for advancing our understanding of synaptic organization and plasticity. However, spine identification remains challenging due to their heterogeneous morphologies, dense clustering, and local curvature variations. Manual annotation is highly time-intensive and impractical for large-scale datasets, underscoring the need for automated and reliable computational methods. Several segmentation methods have been proposed in recent years [3, 4, 32, 36, 38, 41, 43, 45, 47], yet challenges remain in achieving both accuracy and scalability.

To address these challenges, we introduce a method for automated spine segmentation that combines discrete differential geometry with deep learning. Our framework begins by preprocessing 3D reconstructions of dendritic segments to smooth the triangulated mesh, reduce noise, and enhance surface quality. From the resulting meshes, we extract geometric descriptors such as Gaussian and mean curvature, along with additional features including distances from the dendritic shaft skeleton and clustering-based descriptors. Together, these features provide a rich representation of both local and global morphology.

Building on this geometric foundation, we developed a series of deep neural network (DNN) architectures to evaluate how feature enrichment impacts segmentation performance. The baseline model, $DNN_{1}$, relies primarily on curvature-based descriptors. $DNN_{2}$ extends this by incorporating distance-to-skeleton features, improving the separation of spines from shafts. Finally, $DNN_{3}$ integrates a set of enriched geometric and topological descriptors, enabling the network to capture subtle morphological variations and complex spine arrangements. This progression of models demonstrates how systematically incorporating new features enhances both training convergence and segmentation accuracy.

## 2. Methods and materials

In this section, we employ differential geometry to design a deep neural network architecture for the segmentation of dendritic spines. As a first step, we analyze how curvature can contribute to the characterization of dendritic morphology. Building on this analysis, we then develop and explore a deep neural network that leverages the geometric properties of dendrites to achieve accurate segmentation.

### 2.1. Dendritic Morphology Analysis Using Discrete Differential Geometry.

Segmentation of dendritic shafts and spines can be guided by their differential geometric properties, particularly Gaussian and mean curvature. These curvature measures capture local shape variations that distinguish the roughly cylindrical shaft from protruding spines. However, raw curvature values obtained directly from EM reconstructions are often noisy due to the sectioning process and mesh irregularities. To address this, we first smooth the dendritic triangulated surface mesh using discrete differential geometry techniques, then compute curvature values, and finally enhance these through image processing filters. This three-step process—smoothing, curvature computation, and enhancement—provides robust geometric descriptors that form the basis for accurate segmentation.

#### 2.1.1. Smoothing the Triangulated Surface Mesh.

To analyze the triangulated surface of the dendritic mesh, we first address the inherent roughness in the 3D images caused by the EM sectioning process. We apply discrete differential geometry techniques, specifically the mean curvature (or Willmore) flow, to smooth triangular surfaces mesh.

Let $n_{j}$ be the normal vector to the triangle formed by the vertices ${\text{X}}_{l}$, $X_{j-1}$, $X_{j}$, with A denoting the area of the triangle, and $E_{l,m}$ representing edge vectors between neighboring vertices $X_{l}$ and $X_{m}$. The discrete mean curvature at vertex $X_{l}$ may be computed as [30, 31, 39]:

(1) $$H=\frac{1}{2A}\sum_{m=j}E_{l,m}\times n_{m+1}-E_{l,m}\times n_{m}.$$

Next, we define $k_{bend}$ as the bending coefficient of the surface. The curvature energy over a surface A is given by:

$$W_{bend}\left(X\right)=\frac{k_{bend}}{2}\int_{A}H^{2}\left(X\right)dA,$$

and the bending force is derived as the negative gradient of this energy:

$$F_{bend}\left(X\right)=-\nabla_{X}W\left(X\right).$$

We smooth the curvature by evolving the surface by a gradient descent:

$$\frac{\partial }{\partial t}X=F_{bend}\left(X\right).$$

**Figure 1.**
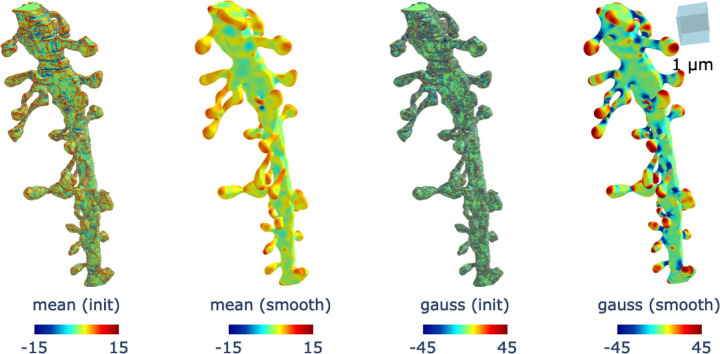
Effect of curvature smoothing on dendritic meshes. The plots labeled (init) and (smooth) show the initial dendritic curvature and the curvature after smoothing, respectively. The plots labeled mean and gauss correspond to the mean curvature and Gaussian curvature of a segment of dendrite. Smoothing enhances the curvature profile, producing a more easily interpretable pattern in the mesh that can be effectively leveraged to improve segmentation accuracy. For visualization, Gaussian curvature values are thresholded to remain within an absolute value of 45, while the mean curvature are constrained within an absolute value of 15, thereby highlighting the most relevant mesh faces.

Further details of the numerical computation, which follows the approach described in [46] are provided in Appendix A. The results of performing this smoothing are illustrated in Figure 1, where we compute the Gaussian and mean curvature of a segment of spiny dendrite. The data was obtained from high-resolution 3D EM reconstructions of the dorsal dentate gyrus of the hippocampus in adult rats that underwent in vivo electrophysiological recordings, as described in [6,7]. Comparing the curvature before and after smoothing, we observe that the processed mesh exhibits a more easily interpretable profile of the dendritic surface, which can be exploited to improve the accuracy of spine-shaft segmentation.

#### 2.1.2. Gaussian and Mean Curvature.

In this section, we motivate the use of Gaussian and mean curvature values as key variables for segmenting the dendritic shaft. In the study of dendrite morphology, we assume that dendritic shafts exhibit an approximately cylindrical shape, from which spines are protrude.

The Gaussian curvature provides insight into the surface’s shape at different points. At hyperbolic points, where the surface curves in opposite directions (saddle-like), the Gaussian curvature is negative. Conversely, at elliptical points, where the surface curves uniformly in the same direction (dome-like), the Gaussian curvature is positive. This contrasts with the cylindrical shaft, along which the Gaussian curvature is zero. Applying these principles to dendritic morphology, we observe that along the spine neck and at the intersection between the neck of a spine and the dendritic shaft, the surface exhibits a saddle shape, leading to negative Gaussian curvature. On the other hand, the spine head, with its more spherical or dome-like structure, exhibits positive Gaussian curvature.

The mean curvature further characterizes the surface. On a concave surface, the mean curvature is positive, while on a convex surface, it is negative. This distinction helps to identify regions of significant shape change. For instance, at the transition from the dendritic shaft to the spine neck, the surface is concave, leading to relatively positive mean curvature. In contrast, the spine head, which is a convex region, has relatively negative mean curvature.

An important enhancement step in the shaft segmentation process involves boosting the dendrite curvature values to improve the segmentation’s accuracy as we discuss next in detail.

#### 2.1.3. Enhancement of Curvature through Image Processing.

Here, we provide an intuition for using image processing techniques to enhance the mean and Gaussian curvature values. The goal is to emphasize regions of the surface with significant curvature changes, which are more likely to correspond to dendritic shafts or spines, thereby facilitating segmentation. This forms the foundation for the machine learning methods that will be developed in the segmentation algorithm.

First, let us define the following sigmoidal function, which is often used in image processing to normalize and enhance contrast:

$$\zeta \left(x\right)=\frac{1}{1+\exp\left(-x\right)}.$$

This transformation maps all real values into the interval $\left(0,1\right)$. For large positive x, ζ(x)→1, while for large negative x, ζ(x)→0. Around x=0, the function has its steepest slope, which enhances small variations near zero and makes them more distinguishable.

Applying this transformation to the mean curvature H and the Gaussian curvature K, we obtain:

(2) $$\tilde{H}=\zeta \left(a_{H}H+b_{H}\right),\;\tilde{K}=\zeta \left(a_{K}K+b_{K}\right),$$

where $a_{H}$, $b_{H}$, $a_{K}$, and $b_{K}$ are empirically chosen parameters used to emphasize specific geometric features of the dendritic mesh. For example, in the neck region of a spine, we expect relatively high negative Gaussian curvature. By appropriately choosing $a_{K}$ and $b_{K}$, these negative values are pushed toward the lower end of the sigmoid, making them stand out more clearly during segmentation.

In contrast, along the cylindrical shaft, the Gaussian curvature is close to zero. Proper tuning ensures that values near zero are mapped consistently with the shaft, so that these regions are correctly identified. For mean curvature, which distinguishes concave and convex regions, the transformation highlights transitions: concave regions (positive mean curvature) are enhanced toward higher sigmoid values, while convex regions (negative mean curvature) are mapped toward lower values.

In practice, within this paper, $\tilde{H}$ and $\tilde{K}$ will not be directly computed. The process of obtaining them merely serves to justify the use of a deep neural network as an approximation function to address the segmentation problem, with the sigmoidal function employed as the output layer in the DNN.

### 2.2. Deep Neural Network Approach for Spine and Shaft Analysis.

In the previous section, we provided an intuitive explanation of how segmentation can be enhanced using image processing techniques. We then introduced empirical filtering parameters that can improve segmentation quality. Instead of manually selecting these parameters and explicitly computing $\tilde{H}$ and $\tilde{K}$, we can leverage Deep Neural Networks (DNNs) to learn an optimal segmentation approximation function based on H and K. At the same time, DNNs allow us to incorporate non-linearities that further enhance segmentation performance.

We analyze three different DNN architectures. The first network is designed to support the second by assisting in the extraction of the shaft skeleton, while the third network relies solely on external Python libraries for skeletonization. Machine learning methods have also been employed for dendrite segmentation of dendritic spines obtained from confocal reconstruction images using Convolution Neural Networks CNNs [43]. In this work, we adopt a simple DNN architecture inspired by the physics-informed neural network (PINN) model [17, 19, 25, 27, 28, 33–35], which has been widely applied to approximate ordinary and partial differential equations. These models leverage their well-known ability to serve as universal function approximators [17]. Given the geometrical characteristics of the dendritic triangular mesh, this approximation capability of DNNs constitutes a fundamental tool in our algorithm. Here, we present the architectures that produced the best results among the different trials.

#### 2.2.1. Deep Neural Network (DNN_1_) using the Gaussian and Mean Curvature.

As for the sigmoidal function (2) introduced in the previous section, various model parameters require fine-tuning to enhance the shaft segmentation process. Rather than manually selecting these parameters and computing $\tilde{H}$ and $\tilde{K}$ we train a deep neural network in a two-step procedure.

**Figure 2.**
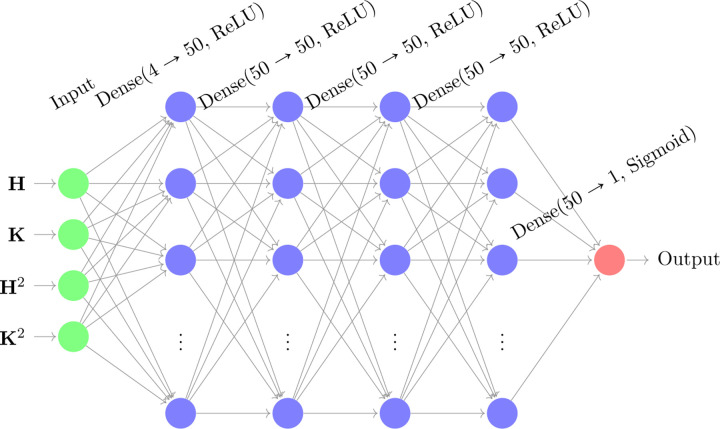
Deep Neural Network ($DNN_{1}$) architecture used the dendrite shaft. The input layer consists of the mean curvature H and the Gaussian curvature K, computed after smoothing, and their squares. The network includes four hidden layers, each with fifty neurons and ReLU activation functions. The output layer employs a sigmoidal activation function.

We first train a deep neural network, denoted as $DNN_{1}$, whose architecture is illustrated in Fig. 2. The input layer receives both the mean curvature H and the Gaussian curvature K values of the dendritic triangular mesh (after smoothing), together with their squared terms to capture higher-order variations. The input features are preprocessed such that the Gaussian curvature values and their squared terms are thresholded to remain within an absolute value of 45. Specifically, for $K=(K_{1},K_{2},...,K_{m})$, we enforce $K_{i}\in [-45,45]$. We then compute $K_{i}^{2}$ and threshold its value to the range [0, 45]. Similarly, the mean curvature values and their squared terms are constrained within an absolute value of 15, following the same procedure as in the Gaussian curvature case.

These thresholds are applied because curvature values can sometimes become very large, and such extreme values do not significantly improve prediction accuracy but can instead cause instability during training and inference. To further reduce instability, any NaN values, if present, are replaced with the average of the corresponding curvature values.

The network consists of four hidden layers, each containing fifty neurons with ReLU activation functions to introduce non-linearity [25]. The output layer employs a Sigmoid activation function, as described in Section 2.1.3, to classify vertices into dendritic shafts and spines.

The segmentation produced by $DNN_{1}$ is not fully satisfactory, as regions within spines are sometimes misclassified as part of the shaft. This misclassification arises because certain spines regions exhibit relatively flat curvature, making them appear similar to shaft regions.

#### 2.2.2. Skeletonization.

In this section, we describe the process used to obtain the skeletonization of dendritic meshes. is an image processing technique that reduces binary shapes to thin, single-pixel-wide lines while preserving their topological structure and connectivity [26, 51].

The first step in building the skeleton is to ensure that the mesh is watertight, meaning that it forms a completely closed surface with no gaps, holes, or disconnected edges. A watertight mesh guarantees a well-defined interior and exterior, which is essential for reliable geometric processing and for preserving the topological structure during skeletonization. To achieve this, we wrap the existing mesh using the algorithm described in Appendix B. This procedure converts the surface into a uniformly sampled point cloud, estimates and orients normals, and then applies Poisson surface reconstruction to generate a closed, watertight representation.

Once a watertight mesh is obtained, we compute its skeleton using the scikit-image skeletonization package, which implements algorithms from [26,51]. Further implementation details are provided in Appendix B. This simplified representation captures the essential branching geometry of dendrites for subsequent segmentation and morphological analysis.

#### 2.2.3. Deep Neural Network ($DNN_{2}$) using Shaft Skeleton.

We can enhance dendritic spine–shaft segmentation by incorporating the shaft skeleton. This involves using the distance between the shaft skeleton vertices and the dendritic branch as an additional input to the DNN. For this purpose, we use the shaft segmented with $DNN_{1}$ together with the skeletonization procedure outlined in Section 2.2.2.

To begin, we consider the shaft-segmented meshes obtained from $DNN_{1}$. Since this mesh results from the dendritic branch after spine regions have been segmented, it is no longer watertight. Therefore, we apply the procedure described earlier to make the mesh watertight. Once the shaft mesh skeletonization is completed, we compute the distance D between the shaft skeleton and the dendritic branch mesh vertices. Here, for each vertex $X_{l}$, $l\in \left\{1,2,...,n\right\}$ in the dendritic triangular mesh with n vertices, and $\left\{V_{1},V_{2},\ldots \;,V_{v}\right\}$ the set of shaft skeleton vertices, we compute the Euclidean norm:

(3) $$D_{l}=\underset{1⩽j⩽v}{\min}{\left\|V_{j}-X_{l}\right\|}_{2},\;D=\left(D_{1},D_{2},\ldots ,D_{n}\right).$$

**Figure 3.**
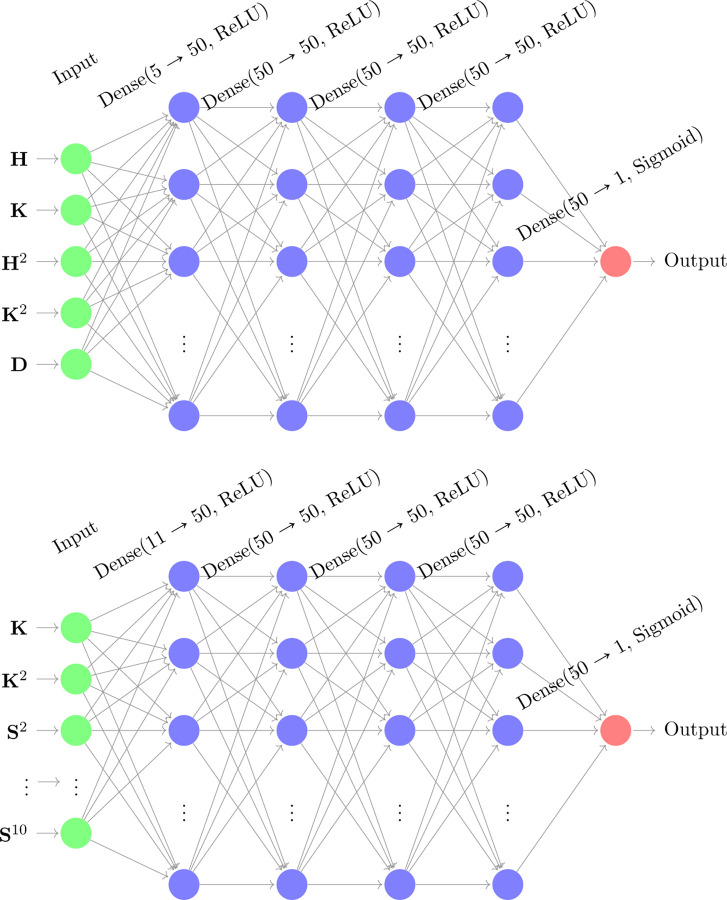
The top diagram illustrates the architecture of the deep neural network ($DNN_{2}$), which improves upon $DNN_{1}$. This model closely resembles the previous network but incorporates an additional input feature: the distance D between the central curve of the shaft (computed using $DNN_{1}$) and the mesh vertices. The output layer employs a sigmoid activation function, consistent with the design of the earlier network. The bottom diagram shows the architecture of the deep neural network ($DNN_{3}$) with additional input features. This model extends the previous design by enriching the input layer with multiple geometric and topological descriptors, including the Gaussian curvature and its squared value, as well as segmentation descriptors $S^{k}$ obtained from K-means clustering of the shortest distances between mesh vertices and the dendrite skeleton. As in the earlier models, the output layer uses a sigmoid activation function.

With this additional information, we develop an improved deep neural network, $DNN_{2}$, whose architecture is shown in Fig. 3. This model is similar to $DNN_{1}$, except that it incorporates the new input feature. As in the earlier model, the input layer receives both the Gaussian and mean curvature values of the dendrite, along with the squared values of these curvatures as additional features. The network consists of four hidden layers, each containing fifty neurons with ReLU activation functions to introduce non-linearity. The output layer employs a Sigmoid activation function, as discussed in Section 2.1.3, to classify vertices into dendritic shafts or spines.

#### 2.2.4. Dendrite Spine-Shaft Segmentation Using Dendritic Branch Regions ($DNN_{3}$).

To improve segmentation accuracy, we introduce a deep neural network ($DNN_{3}$) that incorporates regional information from dendritic branch segments. These segments are derived from the dendrite skeleton, which serves as a structural reference for spatial organization.

For this model, once the skeleton is extracted, we compute the shortest distance from each mesh vertex to its nearest skeleton point. These distances are then clustered using the K-means algorithm to partition the dendritic mesh into multiple regions. This regional segmentation enables us to distinguish between different parts of the dendrite shaft, spine neck, and spine head—regions that are critical for accurate classification.

To capture variations in dendritic morphology, we apply K-means clustering with multiple values of k. We denote these segmentation features by $S^{k}$, where k∈{2,3,…,10} corresponds to the number of clusters. In particular, the value of each feature $S^{k}$ is given by the corresponding cluster label. Therefore this model incorporates 9 additional scalar features. In this study, we explore values of k ranging from 2 to 10 to provide a multi-scale representation of regional structure.

In addition to these region-based segmentation features, we incorporate Gaussian curvature and its squared value as geometric descriptors. These measures are particularly effective for identifying neck regions, as discussed in earlier sections. We intentionally exclude mean curvature as we have observed that the mean curvature input increases the probability of misclassifying all dome-like regions as spines, leading to false positives. By omitting mean curvature and focusing on more discriminative features, $DNN_{3}$ achieves improved segmentation performance compared to the baseline models.

### 2.3. Loss Function.

We use the binary cross-entropy (BCE) to compute the loss function, and its derivation is presented below.

Let us consider a set of m dendritic meshes, denoted as $𝓓=({𝓓}_{1},{𝓓}_{2},...,{𝓓}_{m})$, where each mesh ${𝓓}_{i}$ consists of $n_{i}$ vertices. For the training set, we assume that each ${𝓓}_{i}$ has a ground-truth classification matrix $Y_{i}\;\in \;{\left\{0,1\right\}}^{n_{i}\times 2}$, which represents the classification of vertices in ${𝓓}_{i}$ in one-hot encoding, such that:

$$Y_{i,j}=\left\{\begin{array}{ll} \left(0,1\right) & \text{if}\;\text{vertex}\;j\;\text{of}\;{𝓓}_{i}\;\text{belongs}\;\text{to}\;\text{the}\;\text{dendritic}\;\text{shaft},\\ \left(1,0\right) & \text{otherwise}. \end{array}\right.$$

We define $Y\;=\;(Y_{1},Y_{2},...,Y_{m})$ as the set of all annotation matrices. Next, we define the set of mean curvature vectors for all dendritic meshes as $H\;=\;\left(H_{1},H_{2},...,H_{m}\right)$ and the set of Gaussian curvature vectors as $K\;=\;\left(K_{1},K_{2},...,K_{m}\right)$. For each mesh, ${𝓓}_{i}$, $j\;\in \;\left\{1,2,\ldots ,m\right\}$, the mean curvature vector is given by $H_{j}=(H_{1,j},H_{2,j},...,H_{n_{j},j})$, and the Gaussian curvature vector by $K_{j}=(K_{1,j},K_{2,j},\cdots ,K_{n_{j},j})$, where $H_{i,j}$ and $K_{i,j}$ denote the mean and Gaussian curvatures of ${𝓓}_{j}$ at vertex i, for $i\in \left\{1,2,\ldots ,n_{j}\right\}$.

Additionally, we define the distance vector $D=\left(D_{1},D_{2},...,D_{m}\right)$, representing the distances between the central curve and the vertices, as well as additional structural descriptors $S^{k}$, where $k\;\in \;\left\{2,3,...,10\right\}$. For each mesh ${𝓓}_{j}$, we write $S_{j}^{k}\;=\;\left(S_{1,j}^{k},S_{2,j}^{k},\ldots ,S_{n_{j},j}^{k}\right)$.

We now define the feature vector for vertex i of mesh j as

$$Z_{i,j}=\left(H_{i,j},H_{i,j}^{2},K_{i,j},K_{i,j}^{2},D_{i,j},S_{i,j}^{2},S_{i,j}^{3},\ldots \right),$$

and the corresponding feature matrix for dendritic mesh ${𝓓}_{j}$ as

$$Z_{j}=\left(H_{j},H_{j}^{2},K_{j},K_{j}^{2},D_{j},S_{j}^{2},S_{j}^{3},\ldots \right).$$

Our objective is to find a function

$$f_{\theta }:Z_{j}\mapsto Y_{j}\in {\left[0,1\right]}^{n_{j}\times 2},\;\forall j\in \left\{1,2,\ldots ,m\right\},$$

that best approximates the ground-truth classification by minimizing the following loss function:

$$𝓛=\underset{\theta }{\min}\left\|f_{\theta }\left(Z\right)-Y\right\|,$$

where $\left\|\cdot \right\|$ denotes an appropriate norm measuring the discrepancy between the predicted and actual classifications. In particular, we use a weighted sum of the binary cross-entropy (BCE), which encourages a more balanced optimization process. The binary cross-entropy formula is given by:

$$\text{BCE}\left(y,\hat{y}\right)=-\left(y\;\log\left(\hat{y}\right)+\left(1-y\right)\log\left(1-\hat{y}\right)\right).$$

The final loss function is defined as:

$${𝓛}_{\theta }=\frac{1}{m}\sum_{j=1}^{m}\left(\frac{1}{n_{j}}\sum_{i=1}^{n_{j}}{\left(\text{BCE}\left(Y_{i,j},f_{\theta }\left(Z_{i,j}\right)\right)\cdot w_{j}\right)}^{2}\right).$$

Here the weights $w_{j}$ is empirically determined.

### 2.4. Spine and Shaft Detection.

After the training step of the algorithm, the next stage of spine–shaft segmentation involves applying the model for classification as well as performing additional post-processing steps to first isolate the shaft and then the spines. In this section, we analyze the processes required to obtain a reliable segmentation.

#### 2.4.1. Grouping Dendritic Mesh Parts into Connected Vertices.

After training the DNN, the first step is to classify the vertices of a given test dendritic mesh into *shaft* and *spine* categories. The resulting classification can then be grouped into connected components of spine vertices and shaft vertices.

As a consequence of the sigmoidal activation function applied to the final layer, the output of the DNN is a probability matrix $\bar{Y}=f_{\theta }\left(Z\right)\;\in {\left[0,1\right]}^{n\times 2}$, where each row corresponds to a vertex and contains the probabilities of that vertex being classified as a *spine*, ${\tilde{𝓧}}_{\text{spine}}$ (first column greater than the second), or a *shaft*, ${\tilde{𝓧}}_{\text{shaft}}$ (second column greater than or equal to the first). Formally, we define:

(4) $$\begin{array}{l} {\tilde{𝓧}}_{\text{spine}}=\left\{X_{i}\in 𝓓|b\bar{Y}\left(i,1\right)>a\bar{Y}\left(i,0\right)\right\},\\ {\tilde{𝓧}}_{\text{shaft}}=\left\{X_{i}\in 𝓓|b\bar{Y}\left(i,1\right)⩽a\bar{Y}\left(i,0\right)\right\}. \end{array}$$

Here a, b are empirical parameters to be set. Next, we describe how to group the predicted dendritic parts—classified as either *spine* or *shaft* —into subgroups of connected components. Let each vertex $X_{i}$ have an associated set of neighboring vertices denoted by ${𝓝}_{i}$:

$${𝓝}_{i}=\left\{X_{j}|X_{j}\;\text{is}\;\text{in}\;\text{the}\;1\text{-ring}\;\text{neighborhood}\;\text{of}\;X_{i}\right\}.$$

We define a *connected group* of vertices ${𝓖}_{i}\;\subseteq \;𝓓$ such that for any pair $X_{p}$, $X_{q}\;\in \;{𝓖}_{i}$, there exists a path of vertices $X_{i1},X_{i2},...,X_{i_{m}}\in {\tilde{𝓧}}_{\text{part}}$ satisfying:

$$X_{i_{1}}=X_{p},\;X_{i_{m}}=X_{q},\;X_{i_{\ell }}\in {𝓝}_{i_{\ell -1}}\;\text{for}\;\text{all}\;\ell =2,\ldots ,m.$$

Here, ${\tilde{𝓧}}_{\text{part}}$ denotes the set of vertices classified as a given part type—either ${\tilde{𝓧}}_{\text{spine}}$ for spines or ${\tilde{𝓧}}_{\text{shaft}}$ for shafts. The subset of *part-classified* vertices within this group is then:

$${\tilde{𝓨}}_{\text{part}}^{i}=\left\{X_{j}\in {\tilde{𝓧}}_{\text{part}}|X_{j}\in {\tilde{𝓖}}_{i}\right\}.$$

Finally, the complete set of part-classified vertices ${\tilde{𝓨}}_{\text{part}}$ can be decomposed into disjoint set of connected vertices, where connectivity is defined by neighborhood overlap:

$${\tilde{𝓨}}_{\text{part}}=\left\{{\tilde{𝓨}}_{\text{part}}^{i}|X_{j}\in {\tilde{𝓧}}_{\text{part}}\right\}.$$

#### 2.4.2. Spine–Shaft Detection.

Using the subgroups defined above of connected components, we now define the segmentation of the dendritic mesh into spines and shaft. Let ${\tilde{𝓨}}_{\text{shaft}}$ denote the set of connected components classified as *shaft*. In practice, some of these components may actually be parts of spines, so it is crucial to separate them from the true shaft set for accurate segmentation.

To achieve this, we identify the largest connected component in ${\tilde{𝓨}}_{\text{shaft}}$ and designate it as the *entire shaft*. All remaining connected components in ${\tilde{𝓨}}_{\text{shaft}}$ are then assumed to belong to spines.

The set of vertices belonging to the shaft is defined as:

$${𝓨}_{\text{shaft}}^{0}=\underset{{\tilde{𝓨}}_{\text{shaft}}^{i}\subset {\tilde{𝓧}}_{\text{shaft}}}{\max}\left|{\tilde{𝓨}}_{\text{shaft}}^{i}\right|,$$

where $\left|\cdot \right|$ denotes the cardinality of the set.

Once the entire shaft is defined, spine segmentation proceeds by removing the shaft vertices from the dendrite vertex set, reclassifying them as spine vertices and then reapplying the connected-component grouping process described in Section 2.4.1 to the remaining vertices. The set of segmented spines is defined as:

$${𝓨}_{\text{spine}}=\left\{{𝓨}_{\text{spine}}^{i}|X_{i}\in {\tilde{𝓧}}_{\text{spine}}\right\},$$

where each individual spine component is given by:

$${𝓨}_{\text{spine}}^{i}=\left\{X_{j}\in 𝓓\backslash {𝓨}_{\text{shaft}}^{0}|X_{j}\in {𝓖}_{i}\right\}.$$

### 2.5. Dataset Description.

This section describes the datasets of dendritic segments used for training and testing our algorithm. To mitigate overfitting, the training and testing sets were independent and originate from different animals. This separation ensures that the model learns generalizable features of dendritic structures rather than memorizing patterns specific to a single specimen.

The training dataset comprises six high-resolution 3D EM reconstructions of the dorsal dentate gyrus in the hippocampus of adult rats. These animals underwent *in vivo* electrophysiological recordings, as described in [6, 7].

For testing, we used data from the axon–spine coupling study, which includes a complete nanoconnectomic 3D reconstruction of hippocampal neuropil obtained via serial EM [15]. This dataset contains four independent annotations—two performed by each of two annotators [2]—providing detailed segmentation of dendritic spines. Out of 151 meshes, we selected 28 spiny dendritic branches for testing based on annotation consensus. Specifically, we included only those meshes where two or more annotators agreed on the presence of at least one dendritic spine. Additionally, for each spine mesh identified by the annotators, we retained only those meshes that were consistently labeled as spines by at least two of the selected annotations. This ensured that the testing set reflected a high-confidence subset of spiny dendritic structures.

It is important to note that some structure exhibiting spine-like morphology were not annotated as spines in the test set for two reasons. First, biologically, the presence of a synaptic area in the spine head is essential for defining a spine. Thus, even if a mesh appears spiny, it is not considered a spine if no synapse is present. Second, some meshes were excluded due to incomplete reconstruction—parts of the spine may have been cut off or lost during imaging.

In contrast, the training dataset does not apply as strict criteria for spine identification as the test set. As a result, the testing dataset introduces additional segmentation challenges that are not reflected in the training data. Our algorithm does not explicitly account for these differences, which may influence performance.

For the training dataset, each spine was stored as an individual .obj file in watertight format. The mesh corresponding to the shaft of each dendritic segment was provided separately, along with the complete mesh of the dendritic segment. Because spine and shaft meshes were stored independently, we wrapped the spines and shaft with a tight mesh from the exterior in order to generate a unified dendritic mesh using the algorithm in Appendix B. We then used the independent spine meshes to label the constituent parts of the new wrapped dendritic mesh. To achieve this, we employed a KD-Tree–based nearest-neighbor search to map spine vertices to the wrapped dendritic mesh. Specifically, for each set of spine vertices in the individual dataset vertices, we queried a KD-Tree built from the wrapped dendritic mesh vertices to identify all branch vertices within a radius threshold $r_{\text{th}}$:

$$\text{vertices\_appr}=\text{list}\left(\text{set}\left(\text{np}.\text{concatenate}\left(\text{kdtree}.\text{query\_radius}\left(\text{vertices},r_{\text{th}}\right)\right)\right)\right).$$

Here, query_radius returns the indices of dendrite mesh vertices that lie within a distance $r_{\text{th}}$ of each spine vertex. By aggregating these indices, we obtain the set of dendrite vertices corresponding to the annotated spine regions on the wrapped mesh. This mapping step allows us to merge the spine and shaft meshes into a single labeled dendritic mesh, which is then used for both training and validation.

## 3. Results

This section presents a comprehensive evaluation of our algorithm’s performance in segmenting dendritic shafts and spines. We first describe the identification of dendritic shafts using geometric properties and a neural network-based approach. We then assess the ability of the algorithm to segment dendritic spines, highlighting both its strengths and limitations.

Figure 7 illustrates the predicted dendritic shafts and spines from datasets from Kasthuri et al. [20], demonstrating that our method successfully identifies spines along the dendritic shaft by leveraging geometric properties such as Gaussian and mean curvature. However, misclassification can occur when the dendritic structure deviates significantly from the idealized cylindrical shape, underscoring the need for further improvements to the method.

To make these methods widely accessible, we have posted an open-source code repository on GitHub: GitHub:curvature-based-dendrite-segmentation.

### 3.1. DNN Prediction Results Analysis.

In this section, we present and analyze the prediction results of the three DNNs, $DNN_{1}$, $DNN_{2}$, and $DNN_{3}$ (Figures 2 and 3). The methodology is outlined in Section 2.2.4, and here we focus on two key evaluation criteria: the training loss and the Jaccard index (Intersection over Union, IoU).

All models were trained using the Adam optimizer, which provides efficient and adaptive gradient-based optimization [23]. To promote generalization and reduce overfitting, both L1 and L2 regularization were applied to the hidden layers. This dual regularization strategy encourages sparsity in the learned weights while penalizing large parameter values [16, 40], thereby improving robustness across diverse inputs. The DNN architecture was built using TensorFlow (v2.16.2) [1], and training was performed for approximately 1500 epochs for each model.

**Figure 5.**
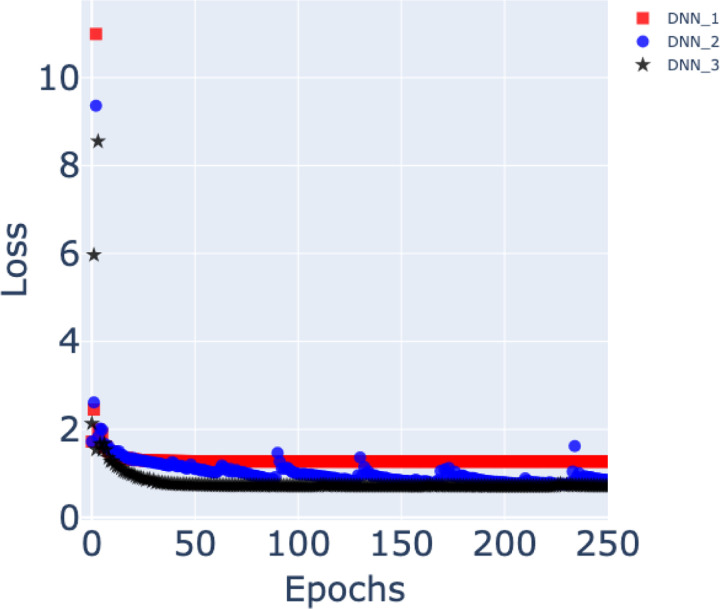
Training loss curves for the three deep neural network models. The red square markers, blue circular markers, and black star markers correspond to the loss curves of $DNN_{1}$, $DNN_{2}$, and $DNN_{3}$, respectively. Among the three models, $DNN_{3}$ achieves the best convergence, reaching a minimum loss of approximately 0.7. In contrast, the basic architecture $DNN_{1}$ shows the poorest convergence behavior, with higher residual loss throughout training. These results highlight the effectiveness of the additional features and architectural refinements introduced in $DNN_{2}$ and $DNN_{3}$ for improving model optimization and stability.

The training loss curves for the three models are shown in Fig. 5. Among the three, $DNN_{3}$ achieves the best convergence, reaching a minimum loss of approximately 0.70. $DNN_{2}$ also converges well, though slightly less effectively than $DNN_{3}$, while the baseline architecture $DNN_{1}$ shows the poorest convergence, with higher residual loss throughout training. These results highlight the importance of the additional features and architectural refinements introduced in $DNN_{2}$ and $DNN_{3}$ for improving optimization stability.

**Figure 6.**
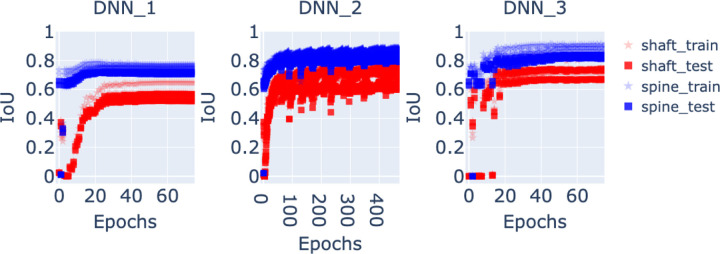
Intersection over Union (IoU) curves obtained during training of the three neural network models. Each plot compares the annotated vertices with the predicted vertices for the dendritic shaft (red curves) and the spines (blue curves). The star markers represent IoU values computed on the training datasets, while the square markers correspond to the testing datasets. Overall, $DNN_{2}$ achieves the best performance, with average IoU values of approximately 0.70 for the shaft and 0.81 for the spines, consistent with its superior loss convergence shown in Fig. 5. In contrast, $DNN_{1}$ performs the worst, with average IoU values of 0.55 for the shaft and 0.75 for the spines. The similarity between training and testing curves indicates that all three models generalize well without significant overfitting.

To quantitatively assess segmentation accuracy, we computed the IoU between predicted and annotated vertices for both the dendritic shaft and spines, on both the training and test sets. The IoU curves are presented in Fig. 6. For $DNN_{2}$, the IoU converges to average values of approximately 0.70 for the shaft and 0.81 for the spines, consistent with its superior loss convergence. $DNN_{3}$ achieves comparable performance, while $DNN_{1}$ lags behind with average IoU values of 0.55 for the shaft and 0.75 for the spines. Importantly, the similarity between training and testing curves across all models indicates good generalization and minimal overfitting.

Overall, these results demonstrate a clear progression in performance from $DNN_{1}$ to $DNN_{3}$. The incorporation of additional geometric and topological features in $DNN_{2}$ and $DNN_{3}$ significantly improves both convergence and segmentation accuracy, underscoring the value of feature enrichment in dendritic spine detection.

### 3.2. Dendritic Spine Detection Analysis.

Following dendritic shaft segmentation, we applied the spine detection algorithm described in Section 2.4 to identify spines. For each detected spine, we computed the IoU to evaluate segmentation accuracy. In addition, we calculated the IoU for the union of all detected spines to assess overall performance. We then computed accuracy, precision, recall, and F1-score to further quantify classification performance.

#### 3.2.1. Qualitative Evaluation.

**Figure 7.**
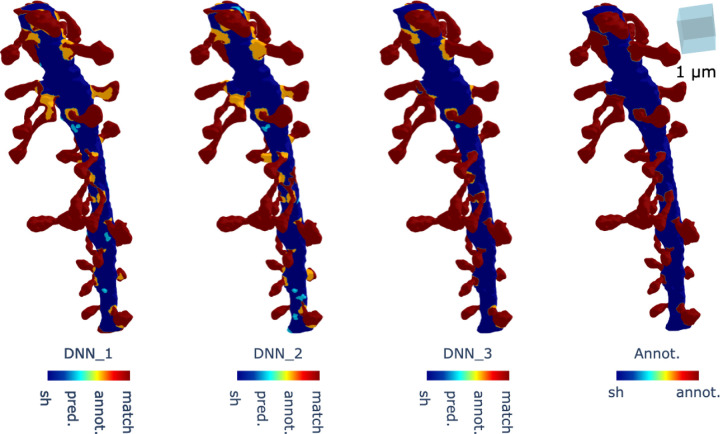
Comparison of segmentation results obtained using $DNN_{1}$, $DNN_{2}$, and $DNN_{3}$. The first three panels show the predicted segmentation results from each network, while the last panel displays the expert spine annotation used as ground truth for evaluation. In the meshes, red vertices indicate correct predictions that match the annotation, sky blue vertices represent shaft regions misclassified as spines, and yellow vertices represent spine regions misclassified as shaft. $DNN_{1}$ produces noisier results with more misclassifications, particularly in clustered regions. $DNN_{2}$ improves segmentation by reducing shaft-to-spine misclassifications, while $DNN_{3}$ demonstrates the most accurate classification overall, with fewer errors and a closer match to the expert annotation.

The segmentation results obtained using the DNN models are shown in Fig. 7. A clear qualitative improvement can be observed when moving from $DNN_{1}$ to the more advanced architectures $DNN_{2}$ and $DNN_{3}$. While $DNN_{1}$ often misclassifies regions of the shaft as spines, leading to fragmented and noisy segmentation, $DNN_{2}$ reduces these errors by incorporating additional geometric features such as the distance to the shaft skeleton. This results in a more coherent representation of the dendritic shaft and a better separation between shaft and spine regions. Finally, $DNN_{3}$ further refines the segmentation by integrating enriched geometric and topological descriptors, which allows it to capture subtle curvature variations and complex spine clusters more effectively. Visually, this is reflected in the reduced number of misclassified vertices and the closer alignment of the predicted segmentation with the expert annotation. These qualitative observations are consistent with the quantitative improvements reported in Tables 1, in which $DNN_{2}$ and $DNN_{3}$ outperform $DNN_{1}$ across all evaluation metrics.

#### 3.2.2. Quantitative Evaluation and Analysis.

We evaluated our algorithm using two complementary strategies. First, we computed the IoU for each detected spine by comparing it to the annotated segmentation. Second, we computed the IoU for the union of all detected spines compared to the union of the annotated spines. This union-based IoU provides a fairer evaluation of complex spine groups, acknowledging that our algorithm is not specifically designed to segment these structures individually. To further assess classification performance, we also computed accuracy, precision, recall, and F1-score for three deep neural network models, denoted as $DNN_{1}$, $DNN_{2}$, and $DNN_{3}$. The results are summarized in Table 1.

Under the per-spine IoU criterion, $DNN_{1}$ achieves an accuracy of 0.580, precision of 0.776, recall of 0.696, and an F1-score of 0.734. While its precision is relatively high, the lower recall indicates that it misses a notable fraction of true spines. $DNN_{2}$ improves upon this baseline, with slightly higher accuracy (0.603), recall (0.732), and F1-score (0.756), suggesting a more balanced performance. $DNN_{3}$ achieves the strongest results under this criterion, with the highest accuracy (0.644), recall (0.767), and F1-score (0.784), demonstrating its effectiveness in capturing spines more reliably.

When evaluated using the union-based IoU, all models show improved performance across metrics. This reflects the fact that aggregating spines into a union provides a fairer evaluation of clustered structures. $DNN_{1}$ improves modestly to an accuracy of 0.605 and F1-score of 0.754. $DNN_{2}$ also benefits, reaching an accuracy of 0.625 and F1-score of 0.752. The most substantial improvement is observed for $DNN_{3}$, which achieves an accuracy of 0.719, recall of 0.867, and an F1-score of 0.837. This indicates that $DNN_{3}$ is particularly effective at capturing spines in dense or clustered regions without sacrificing precision.

Overall, these results demonstrate a clear progression in segmentation quality from $DNN_{1}$ to $DNN_{3}$. While $DNN_{1}$ provides a reasonable baseline with strong precision but weaker recall, $DNN_{2}$ offers a more balanced trade-off, and $DNN_{3}$ consistently outperforms both models. The union-based evaluation highlights that $DNN_{3}$ is especially well-suited for handling complex spine groups, offering the best overall balance between precision and recall.

### 3.3. Dendrite Spine Segmentation without Smoothing.

In the segmentation of dendrite triangular meshes, curvature smoothing is typically required. However, when the number of vertices is large—more than 200,000—the process becomes computationally expensive, taking more than 20 hours to produce a smooth mesh. We note that by using $DNN_{3}$, the computational time can be reduced by performing segmentation without smoothing, while still achieving a level of accuracy comparable to that obtained with smoothing.

We evaluated the effectiveness of curvature smoothing with $DNN_{3}$ on both the training and test datasets and compared the accuracy. The computed accuracy for the test dataset is 0.658 (with and without smoothing), while for the training dataset it is 0.704 with smoothing and 0.780 without smoothing. These results show that smoothing the curvature of the triangular mesh when using $DNN_{3}$ is unnecessary, and segmentation may even perform better without it.

This phenomenon may be due to the fact that the nine region-based segmentation features $S^{k}$ enable the DNN to detect the neck area without requiring an enhanced curvature profile produced by smoothing. When we tested the same process on other DNN models, the accuracy was zero in both cases.

### 3.4. Application to Dendritic Surface Meshes with Many Vertices.

We next use our algorithm to segment the spines of a dendritic mesh dataset containing a large number of vertices and multiple branches.

In particular, the dataset we study is from the reconstruction of a sub-volume of mouse neocortex [20]. We analyzed a dendritic mesh with 5,387,879 vertices and 10,777,005 faces. This dendritic mesh is about 13.42 times larger than the training dataset used in the previous sections (401,371 vertices and 802,750 faces). While most of the training and testing datasets contain only one main dendritic branch with attached spines, this dataset has nine branches with multiple spines. This highlights how much more complex this dataset is compared to the training and testing data.

Because this dataset is relatively large, the segmentation process differs slightly from that used in training. Computations were performed on a MacBook Pro with an Apple M4 Max chip and 64 GB of memory. When we attempted to segment the large dendritic mesh directly, the process failed due to computational constraints. Therefore, we reduced the size of the mesh using the simplification algorithm described in Appendix B. The mesh was simplified to nearly the size of the largest training dataset, given (about 449,109 vertices and 900,000 faces), and we then proceeded with the same testing process as before.

The segmentation results are shown in Figure 9. We observe that $DNN_{3}$ achieves the best performance, correctly labeling most spines based on visual inspection. However, it mislabels a fraction of one sub-branch as spines, as seen in the bottom panel (C). Overall, most spines are well classified, and the sub-branch misclassification may be due to the proximity of the sub-branch mesh to the skeleton at the branching point.

In contrast, $DNN_{1}$ also performs well, correctly identifying most spines and rarely mislabeling sub-branches as spines. Closer inspection of the bottom panel (A) shows that $DNN_{1}$ often misclassifies spine necks as shaft, and in some cases entire spine meshes were mislabeled as shaft. This limitation may arise from the lack of spatial information, as Gaussian and mean curvature alone provide insufficient cues for accurate segmentation.

Finally, $DNN_{2}$ demonstrates the weakest performance. It correctly labels spines only on one of the nine sub-branches, failing on the remainder. This poor performance may be attributed to the shaft–skeleton distance, Gaussian curvature, and mean curvature features being insufficient to reliably identify spine neck regions in this complex dataset.

### 3.5. Dendritic Spine Morphologic Parameters.

In this section, we use the segmentation results obtained from our algorithm to compute several morphological parameters of the dendritic meshes in both the training and testing datasets. These parameters include the neck diameter, head diameter, and spine length.

The computation of head and neck diameters was performed using the spine skeleton. This skeleton was obtained by identifying the closest point of the triangular mesh skeleton to each vertex of the segmented spine mesh. To determine the neck and head diameters, we considered each segmented spine’s triangular mesh. For each vertex, we computed the shortest distance to its nearest skeleton point, as described in equation 3, while computing D. This procedure yielded a thickness profile along the spine. The neck radius was then defined as the minimum of these computed averages, starting from the skeleton vertices closest to the dendritic shaft. Conversely, the head diameter was defined as the maximum computed distance, measured from the skeleton vertices at the farthest point from the shaft.

Additionally, to compute the length of the spine, we used the spine skeletonization and interpolated additional points along the skeleton using a spline technique. This interpolation increases the accuracy of the computed distances by smoothing and interpolating along the dendritic spine skeleton [9, 10, 44]. Additional details are provided in Appendix C. The spine length was then computed as the sum of Euclidean distances between consecutive interpolated points:

$$L=\sum_{i=1}^{N-1}{\left\|V_{i+1}-V_{i}\right\|}_{2},$$

where $V_{i}$ denotes the i-th interpolated vertex.

**Figure 8.**
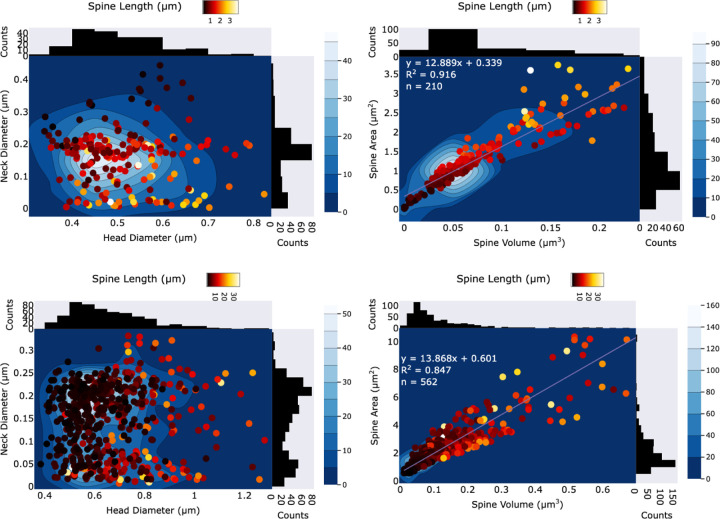
Distribution of morphological parameters in the training dataset and in the large dendritic mesh dataset, obtained using $DNN_{3}$. In the first column, head diameter is plotted against neck diameter of dendritic spines, while in the second column spine volume is plotted against spine area. A colormap encodes spine length. The segmented training dataset contains 210 spines, with an average neck diameter of 0.166 ± 0.0969 *µ*m, an average head diameter of 0.512 ± 0.101 *µ*m, and an average spine length of 1.35 ± 0.694 *µ*m. The average spine volume is 0.122 ± 0.121 *µ*m^3^ and the average spine area is 2.30±1.83 *µ*m^2^, yielding an average area-to-volume ratio of 12.9 with a coefficient of determination *R*^2^ = 0.916. The segmented large dendritic mesh dataset contains 562 spines, with an average neck diameter of 0.148 ± 0.0787 *µ*m, an average head diameter of 0.661 ± 0.164 *µ*m, and an average spine length of 5.57 ± 5.34 *µ*m. The average spine volume is 0.090 ± 0.085 *µ*m^3^ and the average spine area is 1.53 ± 1.15 *µ*m^2^, yielding an average area-to-volume ratio of 13.9 with a coefficient of determination *R*^2^ = 0.847.

**Figure 9.**
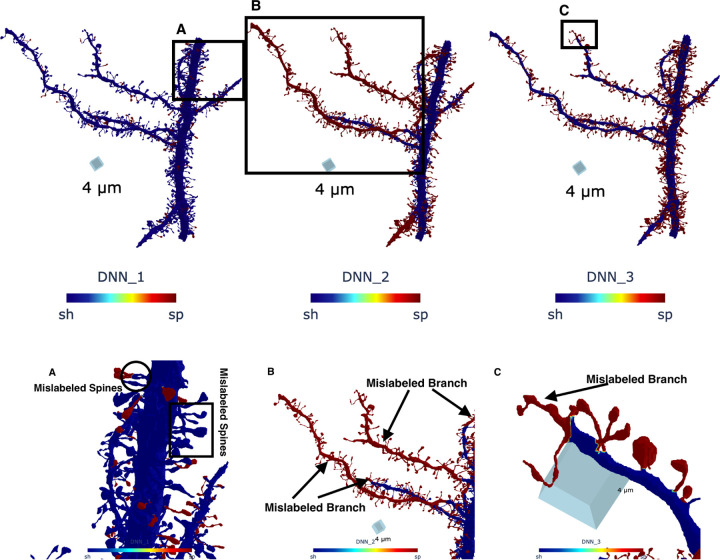
Comparison of segmentation results obtained using $DNN_{1}$, $DNN_{2}$, and $DNN_{3}$ on a large dendritic mesh from [20]. In the figure, blue indicates the shaft mesh (denoted as sh), while red highlights the segmented spines (denoted as sp). The top panel shows that $DNN_{1}$ and $DNN_{3}$ achieve the most accurate segmentation, correctly labeling the majority of spines across the dendritic mesh. In contrast, $DNN_{2}$ incorrectly labels nine subbranches of the dendrite segment as spines. Three boxed regions (A–C) in the top panel highlight misclassified areas, which are shown in detail in the corresponding bottom panels. Panel (A) reveals spine meshes mislabeled as shaft by $DNN_{1}$ (indicated by the box and circle). Panels (B) shows dendritic subbranches mislabeled as spines by $DNN_{2}$, while Panel (C) illustrates similar mislabeling by $DNN_{3}$.

The distribution of morphological parameters in the training and large dendritic mesh datasets is shown in Figure 8. In the first column, the head diameter is plotted against the neck diameter of dendritic spines, while in the second column the spine volume is plotted against the spine area, with a colormap encoding spine length. For clearer visualization, some outliers were removed. Marginal histograms of head and neck diameters are also included to facilitate interpretation.

Quantitatively, the segmented training dataset contains 210 spines, with an average neck diameter of 0.166±0.0969 *µ*m, an average head diameter of 0.512±0.101 *µ*m, and an average spine length of 1.35 ± 0.694 *µ*m. The average spine volume is 0.122 ± 0.121 *µ*m^3^ and the average spine area is 2.30 ± 1.83 *µ*m^2^, yielding an average area-to-volume ratio of 12.9. Importantly, the relationship between spine volume and area is very strong, as reflected by a high coefficient of determination (*R*^2^ = 0.916).

In contrast, the segmented large dendritic mesh dataset contains 562 spines, with an average neck diameter of 0.148 ± 0.0787 *µ*m, an average head diameter of 0.661 ± 0.164 *µ*m, and an average spine length of 5.57±5.34 *µ*m. The average spine volume is 0.090±0.085 *µ*m^3^ and the average spine area is 1.53 ± 1.15 *µ*m^2^, yielding an average area-to-volume ratio of 13.9. Here too, the relationship between spine volume and area remains strong, with a coefficient of determination of *R*^2^ = 0.847.

## 4. Discussion

**Figure 10.**
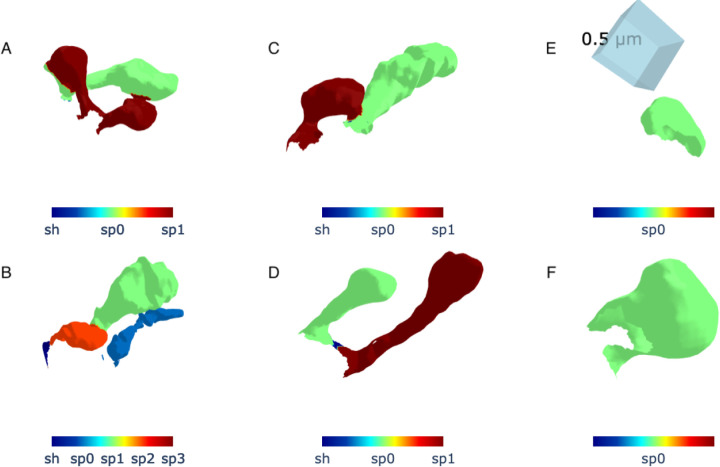
Examples of misclassification encountered during spine segmentation. In the figure, blue indicates the shaft mesh (denoted as sh), while other colors indicate spine meshes (denoted as $sp_{i}$) that were misclassified. Panels (A–D) illustrate cases where multiple spines were incorrectly merged and classified as a single spine. Panel (E) depicts a region of the dendritic shaft that was mistakenly labeled as a spine, while Panel (F) shows a fraction of a spine that was only partially labeled. These examples highlight the challenges of accurately separating spines in dense clusters.

Despite its overall promise, our algorithm encounters challenges in segmenting spines with complex morphologies and spatial arrangements. Below, we discuss three key failure cases, illustrated in Figures 10. The consistently high precision suggests that when a spine is detected, it is almost always correct (few false positives). However, the moderate recall for IoU alone indicates that some spines are missed (false negatives), potentially affecting downstream morphological analyses. The substantial performance improvement with union-based IoU suggests that a more inclusive segmentation strategy enhances detection reliability.

### 4.1 Limitations in Spine Group Segmentation.

Dendritic spines frequently appear in dense clusters, complicating their segmentation. These spine groups are two or more spines whose vertices are directly connected, and after the shaft is removed they still group together. Figure 10 shows a region containing tightly packed spines where the algorithm struggles to distinguish individual structures due to overlapping curvature features. This limitation highlights the need for improved clustering-based segmentation techniques or the integration of additional geometric descriptors capable of differentiating individual spines in high-density regions.

#### 4.1.1. Misclassification of Dendritic Shaft Regions as Spines.

Another failure mode involves the erroneous classification of dendritic shaft regions as spines, as depicted in Figure 10[F]. This misclassification likely stems from local curvature variations that resemble spine-like features. To mitigate this issue, we propose the incorporation of spatial continuity constraints, contextual neighborhood information, or multi-scale curvature analysis to better distinguish dendritic shafts from true spine structures.

#### 4.1.2. Scalability and Large Dataset Limitations.

A further limitation arises from the scale of the datasets used in training and evaluation. High-resolution 3D EM reconstructions of dendritic segments generate extremely large meshes, often containing millions of vertices. Processing such datasets requires substantial computational resources, both in terms of memory and runtime. This constraint limits the feasibility of applying our method to very large-scale reconstructions or to entire brain regions without significant preprocessing or downsampling. Moreover, the need to balance mesh resolution with computational efficiency may lead to the loss of fine structural details, particularly in thin spine necks or small protrusions. Addressing this limitation will require the development of more efficient algorithms, parallelized implementations, or hierarchical multi-resolution approaches that can scale to increasingly large datasets while preserving biologically relevant detail.

In this work, we developed and evaluated three deep neural network architectures for dendritic shaft and spine segmentation. Our results demonstrate a clear progression in performance from the baseline $DNN_{1}$ to the improved models $DNN_{2}$ and $DNN_{3}$. The incorporation of additional geometric and topological features significantly improved both training convergence and segmentation accuracy, as reflected in lower loss values and higher IoU scores. In particular, $DNN_{2}$ achieved the most stable loss convergence, while $DNN_{3}$ provided the best balance between precision and recall, especially in complex clustering scenarios. Notably, $DNN_{3}$ requires only a single training process, compared to the two-stage training of $DNN_{2}$, yet achieves nearly the same level of accuracy, making it a more efficient alternative.

Despite the promise of this approach, challenges remain in accurately segmenting spines with dense spine groups, sub-branches, or local curvature variations that resemble shaft regions. Addressing these limitations will require further refinement of network architectures, the integration of richer geometric and contextual descriptors, and the development of post-processing strategies to merge fragmented predictions. In addition, scaling to even larger datasets presents computational challenges that must be addressed through more efficient algorithms, parallelization, or multi-resolution approaches.

Overall, our findings highlight the potential of feature-enriched deep learning approaches for robust dendritic spine detection. By improving segmentation accuracy and generalization, these models provide a stronger foundation for downstream morphological analyses and quantitative studies of synaptic connectivity. In the long term, such advances will contribute to a deeper understanding of neuronal circuit organization and the structural basis of brain function.

## Figures and Tables

**Figure 4.**
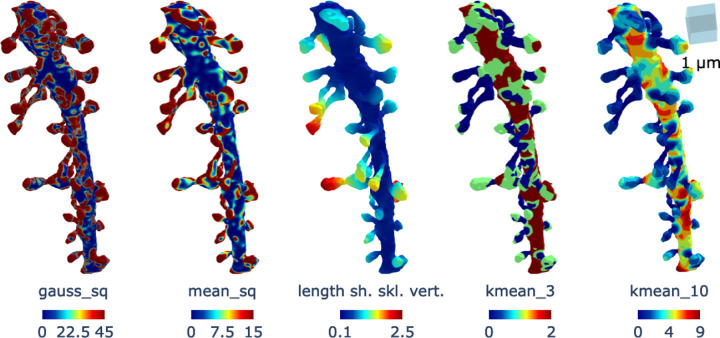
Additional features used in the DNN training. The plot labeled gauss_sq and mean___sq show respectively the square of the Gaussian and mean curvature of the dendrite. The plot labeled length sh. skl. vert. indicates the distance from the shaft skeleton to the dendritic mesh vertices, denoted by D. The plot labeled ${\text{kmean}}_{\text{\_}}k$ illustrates the k-subdivision from K-means clustering, corresponding to the set $S^{k}$.

**Figure 11.**
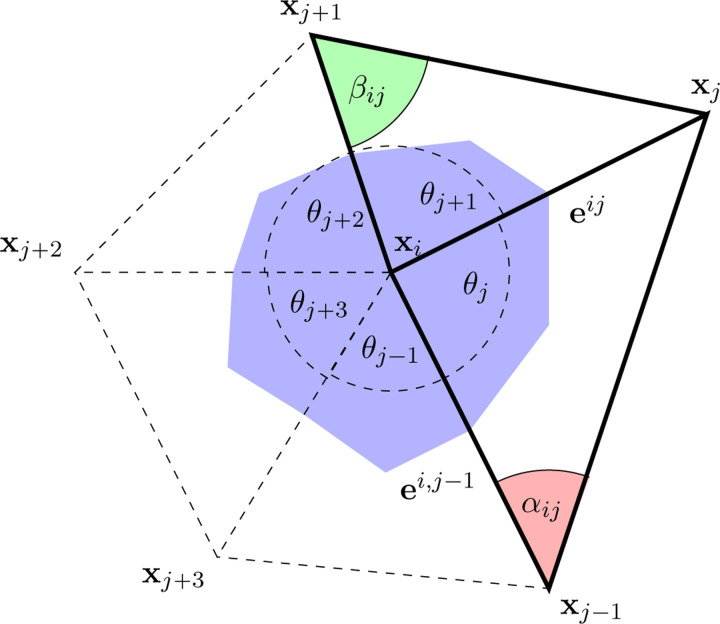
The graph shows the five triangular meshes of 1-ring neighbors to the point $p=X_{i}$. It also depicts the incident angles $\alpha_{ij}$ and $\beta_{ij}$ opposite to the edge $E_{ij}$. The blue area constitutes the barycentric area $A_{p}$.

**Table 1.** Performance metrics for three deep neural network models ($DNN_{1}$, $DNN_{2}$, and $DNN_{3}$) evaluated on dendritic spine segmentation using two criteria: per-spine Intersection over Union (IoU) and Union of IoU. Under the IoU criterion, $DNN_{3}$ achieves the best overall performance with the highest accuracy (0.658) and F1-score (0.794), followed by $DNN_{2}$ (accuracy 0.603, F1-score 0.756) and $DNN_{1}$ (accuracy 0.580, F1-score 0.734). When evaluated with the Union of IoU, all models show improved results, with $DNN_{3}$ again outperforming the others (accuracy 0.721, F1-score 0.838). Precision remains consistently high across models and criteria, indicating a low incidence of false positives. These results highlight that the Union of IoU provides a more comprehensive and biologically meaningful assessment of segmentation quality, particularly in dense dendritic environments.

Model	Criterion	Accuracy	Precision	Recall	F1-score
$DNN_{1}$	IoU	0.580	0.776	0.696	0.734
	Union of IoU	0.605	0.784	0.726	0.754
$DNN_{2}$	IoU	0.603	0.774	0.732	0.756
	Union of IoU	0.625	0.780	0.759	0.752
$DNN_{3}$	IoU	0.658	0.803	0.785	0.794
	Union of IoU	0.721	0.817	0.860	0.838

## Appendix

### Appendix A. Discrete Gaussian and Mean Curvature

In this section, we briefly review the discrete differential geometry formulation presented in [30, 31, 39, 46] to derive the Gaussian and the mean curvature of triangular meshes. Assume the mesh has 𝒱 vertices and 𝓣 triangular faces. Each triangular face ${𝓣}_{l}\in 𝓣$ has vertices denoted by $k_{i}^{l}$, $k_{i+1}^{l}$ and $k_{i+2}^{l}$. These vertices are indexed in a counterclockwise order around the face, defined as follows:

Let $k_{\text{next}}(\cdot )$ denote the next vertex in the counterclockwise direction from ⋅. Then:

- $k_{i}^{l}$ is the first vertex.
- $k_{i+1}^{l}=k_{\text{next}}\left(k_{i}^{1}\right)$ is the next vertex in the counterclockwise direction.
- $k_{i+2}^{l}=k_{\text{next}}\left(k_{i+1}^{1}\right)$ is the vertex following $k_{i+1}^{l}$ in the counterclockwise direction. Then $k_{i+2}^{l}=k_{\text{next}}\left(k_{\text{next}}\left(k_{\text{next}}\left(k_{i}^{l}\right)\right)\right)$.
- $k_{i}^{l}=k_{\text{next}}\left(k_{i+2}^{l}\right)=k_{\text{next}}\left(k_{\text{next}}\left(k_{\text{next}}\left(k_{i}^{l}\right)\right)\right)$ completes the cycle.

This leads to the vertex indices cycling according to:

$$k_{i}^{l}=k_{\left(i-1\;\mathrm{mod}3\right)+1}^{l},$$

where (i mod 3) + 1 cycles through the indices {1,2,3}. Moreover, we will denote the set of indices of the 1-ring neighbors of a vertex with index k by $N_{k}$. This set includes the indices of the vertices whose edges are connected to the vertex $X_{k}$:

$${𝓝}_{k}\;=\;\left\{j|\text{there}\;\text{exists}\;\text{an}\;\text{edge}\;\text{between}\;X_{k\;}\text{and}\;X_{j}\right\}.$$

#### A.1. Discrete Gaussian Curvature.

Let us consider an infinitesimal area 𝓐 and denote its diameter by diam $\left(𝓐\right)$. Additionally, let ${𝓐}^{𝓖}$ denote the area of the image of the Gauss map associated with 𝓐. We can express the discrete Gaussian curvature, ${\hat{\kappa }}_{G}$ at a vertex $p=X_{l}$ as:

(5)
$${\hat{\kappa }}_{G}=\frac{1}{𝓐}\iint_{𝓐}\kappa_{G}dA=\frac{1}{𝓐}\sum_{p\in 𝓐}K_{p},\;\text{with}\;K_{p}=2\pi -\sum_{j\in {𝓝}_{i}}\theta_{j}$$

where $\theta_{j}$ are the interior angles at $p=X_{i}$ of the triangles meeting there. The term $K_{p}$ is known as the defect angle at p. Considering vertices $X_{j}$ in the 1-ring neighborhood of $p=X_{l}$, see Fig 11, the angle $\theta_{j}$ can be computed as:

$$\cos\theta_{j}=\frac{\left(X_{j-1}-X_{l}\right)\cdot \left(X_{j}-X_{l}\right)}{\left\|\left(X_{j-1}-X_{l}\right)\cdot \left(X_{j}-X_{l}\right)\right\|}=\frac{E_{l,j-1}\cdot E_{ij}}{\left\|E_{l,j-1}\cdot E_{ij}\right\|},$$

where $E_{i,j}=X_{j}-X_{l}$ is the edge of the vertices $X_{i}$, $X_{j}$.

#### A.2. Mean curvature.

The discrete mean curvature, ${\hat{\kappa }}_{H}$, at a vertex p is defined as:

$${\hat{\kappa }}_{H}=\frac{1}{𝓐}\iint_{𝓐}\kappa_{H}dA=\frac{1}{𝓐}\sum_{p\in 𝓐}H_{p},\;2H_{p}=\iint_{𝓐}\kappa_{H}dA.$$

To compute $H_{p}$, we consider two equivalent methods [30,31,39,46]. First, assuming vertices adjacent to *p* in cyclic order are $X_{j},X_{j+1},\ldots X_{j+n}$. we have:

$$\begin{array}{l} 2H_{p}=\sum_{m=j}E_{l,m}\times n_{m+1}-E_{l,m}\times n_{m}\\ \;\;=-\sum_{m=j}E_{l,m}\times n_{m}, \end{array}$$

where $n_{j}$ is the normal vector to the triangle with vertices $X_{l}$, $X_{j-1}$, $X_{j}$:

$$n_{j}=\frac{\left(X_{j-1}-X_{l}\right)\times \left(X_{j}-X_{l}\right)}{\left\|\left(X_{j-1}-X_{l}\right)\times \left(X_{j}-X_{l}\right)\right\|}=\frac{E_{l,j-1}\times E_{l,j}}{\left\|E_{l,j-1}\times E_{l,j}\right\|}.$$

Alternatively, the second method involves the integral of the Laplace-Beltrami operator [30]:

$$2H_{p}=\sum_{j\in {𝓝}_{i}}\left(\cot\alpha_{l,j}+\cot\beta_{l,j}\right)\left(X_{l}-X_{j}\right),$$

where $\alpha_{l,j}$ and $\beta_{l,j}$ are the angles opposite edge $E_{l,j}$ in the two incident triangles, as shown in Fig 11.

#### A.3. Vector Area.

In this section, we compute the area 𝓐 as in (1), (5), the sum of all the regions that contain p,

$$𝓐=\sum_{p\in 𝓐}A_{p}.$$

To do this, $A_{p}$ is computed using either the conic area or the barycentric formula that we will describe below. Both methods are equivalent in any case [30].

##### A.3.1. Conic area.

The formula used to compute the vector area is:

$$A=\frac{1}{2}\iint_{𝓐}ndA,$$

and specifically in the triangle with edge $E_{i}$, $E_{j}$ case, the area is given by:

$$A_{i,j}=\frac{1}{2}\left\|E_{i}\times E_{j}\right\|$$

As the conic area is equal to the third of the area in the 1-ring neighborhood of p, we have:

$$A_{p}=\frac{1}{3}A=\frac{1}{6}\sum_{j\in {𝓝}_{1}\left(i\right)}\left\|E_{i,j-1}\times E_{ij}\right\|$$

##### A.3.2. Barycenter Area.

To compute the barycentric area (represented in blue in Fig 11), we first find the centroid $C_{ij}$ of each triangle in the 1-ring neighborhood:

$$C_{ij}=\frac{1}{3}\left(X_{i}+X_{j-1}+X_{j}\right).$$

Next, we compute the middle point $X_{ij}$ of each edge $X_{i}$, $X_{j}$:

$$M_{ij}=\frac{1}{2}\left(X_{i}+X_{j}\right).$$

Then the barycentric area corresponding to the triangle with the vertices $X_{i}$, $X_{j-1}$, $X_{j}$ is

$$A_{ij}=\frac{1}{2}\left\|\left(M_{i,j-1}-C_{ij}\right)\times \left(X_{i}-C_{ij}\right)\right\|+\frac{1}{2}\left\|M_{ij}-C_{ij}\right\|\times \left(X_{i}-C_{ij}\right).$$

Thus, the barycentric area of the 1-ring neighborhood $A_{p}$ is:

$$A_{p}=\sum_{j\in {𝓝}_{1}\left(i\right)}A_{i,j}.$$

### Appendix B. Skeletonization

In this section, we describe the skeletonization algorithm applied to a 3D mesh using Python packages such as trimesh, open3d, and scikit-image. All experiments were performed in a Python 3.9.6 environment with the following package versions: numpy (v2.2.6) [13], open3d (v0.18.0) [52], trimesh (v4.6.8) [8], and scikit-image (v0.24.0) [42]. The goal of this algorithm is to extract a simplified, one-voxel-wide medial axis from a complex dendritic mesh, which can then be used for further geometric analysis and as input to the DNN.

To perform skeletonization, the first step is to ensure that the mesh is watertight. A watertight mesh is a closed surface with no gaps, holes, or disconnected edges, which guarantees a well-defined interior and exterior. If the input mesh is not watertight, we apply a wrapping procedure based on Poisson surface reconstruction to generate a closed representation. Once the watertight mesh is obtained, the pipeline proceeds through mesh simplification, voxelization, skeleton extraction, and vertex-to-skeleton mapping.

#### Mesh Wrapping.

To ensure the mesh is watertight and suitable for skeletonization, we apply a wrapping procedure based on Poisson surface reconstruction [21, 22, 52]. This step converts the input mesh into a uniformly sampled point cloud, estimates and orients normals, and then reconstructs a closed surface. The implementation is shown below:

Mesh = trimesh.Trimesh(vertices=vertices, faces=faces)
o3d_mesh = o3d.geometry.TriangleMesh()
o3d_mesh.vertices = o3d.utility.Vector3dVector(mesh.vertices)
o3d_mesh.triangles = o3d.utility.Vector3iVector(mesh.faces)
# Sample points uniformly from the surface
pcd = o3d_mesh.sample_points_poisson_disk(
number_of_points=number_of_points
)
# Estimate and orient normal
pcd.estimate_normals(
search_param=o3d.geometry.KDTreeSearchParamHybrid(
radius=radius, max_nn=max_nn
)
)
pcd.orient_normals_consistent_tangent_plane(k=10)
# Reconstruct a watertight mesh using Poisson surface reconstruction
mesh_poisson, _ = o3d.geometry.TriangleMesh.create_from_point_cloud_poisson(
pcd, depth=8
)
vertices = np.asarray(mesh_poisson.vertices)
faces = np.asarray(mesh_poisson.triangles)

Here, we first convert the input vertices and faces into a trimesh.Trimesh object and then into an Open3D TriangleMesh. From this mesh, we generate a uniformly sampled point cloud using Poisson disk sampling [49, 52] Normals are estimated and consistently oriented to ensure correct surface reconstruction. Finally, Poisson surface reconstruction produces a watertight mesh, which is returned as arrays of vertices and faces for subsequent processing.

#### Mesh Simplification.

We apply a mesh simplification algorithm to reduce the size of the mesh in cases where the number of vertices is very large. This step is crucial for lowering computational complexity and removing unnecessary geometric detail that may interfere with accurate skeletonization. The simplification is performed using quadric decimation [12, 52] in Open3D, as shown in the following code:

Mesh = trimesh.Trimesh(vertices=vertices, faces=faces)
o3d_mesh = o3d.geometry.TriangleMesh()
o3d_mesh.vertices = o3d.utility.Vector3dVector(mesh.vertices)
o3d_mesh.triangles = o3d.utility.Vector3iVector(mesh.faces)
# Simplify the mesh using quadric decimation
o3d_mesh = o3d_mesh.simplify_quadric_decimation(
target_number_of_triangles=target_number_of_triangles
)
# Convert back to a trimesh object for compatibility
mesh = trimesh.Trimesh(
vertices=np.asarray(o3d_mesh.vertices),
faces=np.asarray(o3d_mesh.triangles)
)

Here, we first create a trimesh.Trimesh object from the input vertices and faces. This mesh is then converted into an Open3D TriangleMesh, which supports advanced mesh processing operations. The simplify_quadric_decimation method reduces the number of triangles while preserving the overall geometry and removing fine details. Finally, the simplified mesh is converted back into a trimesh object for compatibility with subsequent steps in the pipeline.

#### Voxelization.

Next, we voxelize the simplified mesh to convert it into a discrete 3D grid representation. This step enables morphological operations such as thinning and skeletonization. The voxelization process is controlled by a resolution parameter, which determines the granularity of the voxel grid:

pitch =(np.median(mesh.edges_unique_length) * 0.25
voxelized = mesh.voxelized(pitch=pitch))
filled = voxelized.fill()
voxels = filled.matrix.astype(bool)

The parameter pitch in mesh.voxelized is determined from the median edge length of the mesh, scaled by a factor of 0.25. This adaptive choice ties the voxel size to the geometric detail of the mesh, ensuring that the discretization captures fine structures without producing an excessively large grid. The fill() method is then applied to close internal cavities, yielding a watertight solid volume. Finally, the voxel grid is converted into a boolean array, where each entry indicates whether a voxel is occupied, providing a suitable representation for subsequent skeletonization.

### Appendix C. Spline-Based Interpolation

Given the set of spine vertices, we apply the splprep function from the scipy.interpolate module in scipy(v0.24.0) [44], which computes a B-spline representation of an *N*-dimensional parametric curve. This enables smoothing and interpolation along the dendritic spine skeleton [9, 10]. The spline is then evaluated using splev to obtain a dense set of interpolated points along the curve:

points = spine_vertices.T
tck, u = splprep([points[0], points[1], points[2]],
s=spline_smooth,
k=max(1, min(3, len(points[0]) - 1)))
x_fine, y_fine, z_fine = splev(np.linspace(0, 1, line_num_points), tck)
interpolated_vertices = np.column_stack([x_fine, y_fine, z_fine])

Here, line_num_points controls the density of interpolation; in our experiments we set it to 150, and the smoothing factor spline_smooth was chosen as 0.03. The resulting interpolated vertices are ordered sequentially along the spline, which is ensured by the spline parameterization itself.

### Appendix D. Confusion Matrix Analysis

We assess the performance of our model by computing standard classification metrics, including accuracy, precision, recall, and the F1-score. This section details the computation process.

To begin, we illustrate the confusion matrix, which provides a detailed comparison between the predicted classifications and the ground truth annotations. This allows for an in-depth evaluation of classification performance. In our framework, a group of vertices is predicted as spine if its IoU with the annotated spine vertices it overlaps exceeds 0.7.

The classification outcomes are defined as follows:

- **True Positives (TP)**: Spines correctly identified by the algorithm.
- **False Negatives (FN)**: Spines present in the ground truth but missed by the algorithm.
- **False Positives (FP)**: Shaft regions incorrectly classified as spines.
- **True Negatives (TN)**: Shaft regions correctly classified.

Once the confusion matrix is computed, we derive the following performance metrics:

Accuracy. Accuracy measures the proportion of correctly classified vertices:

$$\text{Accuracy=}\frac{\text{TP+TN}}{\text{TP+FP+FN+TN}}.$$

Precision.

Precision evaluates the reliability of spine predictions:

$$\text{Precision}=\frac{\text{TP}}{\text{TP}+\text{FP}}.$$

Recall.

Recall measures the model’s ability to detect actual spines:

$$\text{Recall}=\frac{\text{TP}}{\text{TP}+\text{FN}}.$$

F1-score.

The F1-score provides a balanced measure of precision and recall:

$$\text{F1-score}=2\times \frac{\text{Precision}\times \text{Recall}}{\text{Precision}+\text{Recall}}.$$

## References

[1] Abadi M., Barham P., Chen J., Chen Z., Davis A., Dean J., Devin M., Ghemawat S., Irving G., Isard M., Kudlur M., Levenberg J., Monga R., Moore S., Murray D. G., Steiner B., Tucker P., Vasudevan V., Warden P., Wicke M., Yu Y., and Zheng X., Tensorflow: a system for large-scale machine learning, in Proceedings of the 12th USENIX Conference on Operating Systems Design and Implementation, OSDI’16, USA, 2016, USENIX Association, p. 265–283.

[2] Bartol J., Thomas M, Bromer C., Kinney J., Chirillo M. A., Bourne J. N., Harris K. M., and Sejnowski T. J., Nanoconnectomic upper bound on the variability of synaptic plasticity, eLife, 4 (2015), p. e10778. 26618907 10.7554/eLife.10778 PMC4737657

[3] Basu S., Saha P. K., Roszkowska M., Magnowska M., Baczynska E., Das N., Plewczynski D., and Wlodarczyk J., Quantitative 3-D morphometric analysis of individual dendritic spines, Sci. Rep., 8 (2018). 10.1038/s41598-018-21753-8 PMC5825014 29476060

[4] Bernal-Garcia S., Schlotter A. P., Pereira D. B., Recupero A. J., Polleux F., and Hammond L. A., A deep learning pipeline for accurate and automated restoration, segmentation, and quantification of dendritic spines, Cell Reports Methods, (2025), p. 101179. 40972567 10.1016/j.crmeth.2025.101179 PMC12570329

[5] Bourne J. and Harris K. M., Do thin spines learn to be mushroom spines that remember?, Current opinion in neurobiology, 17 (2007), pp. 381–386. 17498943 10.1016/j.conb.2007.04.009

[6] Bowden J. B., Abraham W. C., and Harris K. M., Differential effects of strain, circadian cycle, and stimulation pattern on LTP and concurrent LTD in the dentate gyrus of freely moving rats, Hippocampus, 22 (2012), pp. 1363–1370. 21853503 10.1002/hipo.20972 PMC3292688

[7] Bromer C., Bartol T. M., Bowden J. B., Hubbard D. D., Hanka D. C., Gonzalez P. V., Kuwajima M., Mendenhall J. M., Parker P. H., Abraham W. C., Sejnowski T. J., and Harris K. M., Long-term potentiation expands information content of hippocampal dentate gyrus synapses, Proc. Natl. Acad. Sci. U. S. A., 115 (2018), pp. E2410–E2418. 29463730 10.1073/pnas.1716189115 PMC5877922

[8] Dawson-Haggerty M. Trimesh: A python library for loading and using triangular meshes. https://trimesh.org, 2019.

[9] Dierckx P., Algorithms for smoothing data with periodic and parametric splines, Computer Graphics and Image Processing, 20 (1982), pp. 171–184.

[10] Dierckx P., Curve and surface fitting with splines, in Monographs on numerical analysis, 1996.

[11] Fiala J. C., Spacek J., and Harris K. M., Dendritic spine pathology: cause or consequence of neurological disorders?, Brain research reviews, 39 (2002), pp. 29–54. 12086707 10.1016/s0165-0173(02)00158-3

[12] Garland M. and Heckbert P. S., Surface Simplification Using Quadric Error Metrics, Association for Computing Machinery, New York, NY, USA, 1 ed., 2023.

[13] Harris C. R., Millman K. J., van der Walt S. J., Gommers R., Virtanen P., Cournapeau D., Wieser E., Taylor J., Berg S., Smith N. J., Kern R., Picus M., Hoyer S., van Kerkwijk M. H., Brett M., Haldane A., Del Río J. F., Wiebe M., Peterson P., Gérard-Marchant P., Sheppard K., Reddy T., Weckesser W., Abbasi H., Gohlke C., and Oliphant T. E., Array programming with NumPy, Nature, 585 (2020), pp. 357–362. 32939066 10.1038/s41586-020-2649-2 PMC7759461

[14] Harris K. and Kater S., Dendritic spines: cellular specializations imparting both stability and flexibility to synaptic function, Annual review of neuroscience, 17 (1994), pp. 341–371. 10.1146/annurev.ne.17.030194.002013 8210179

[15] Harris K. M., Spacek J., Bell M. E., Parker P. H., Lindsey L. F., Baden A. D., Vogelstein J. T., and Burns R., A resource from 3d electron microscopy of hippocampal neuropil for user training and tool development, Scientific Data, 2 (2015), p. 150046. 26347348 10.1038/sdata.2015.46 PMC4555877

[16] Hoerl A. E. and Kennard R. W., Ridge regression: Biased estimation for nonorthogonal problems, Technometrics, 12 (1970), p. 55.

[17] Hornik K., Stinchcombe M., and White H., Multilayer feedforward networks are universal approximators, Neural Networks, 2 (1989), pp. 359–366.

[18] Kandel E. R., Dudai Y., and Mayford M. R., The molecular and systems biology of memory, Cell, 157 (2014), pp. 163–186. 24679534 10.1016/j.cell.2014.03.001

[19] Karniadakis G., Kevrekidis Y., Lu L., Perdikaris P., Wang S., and Yang L., Physics-informed machine learning, Nature Reviews Physics, (2021), pp. 1–19.

[20] Kasthuri N., Hayworth K., Berger D., Schalek R., Conchello J., Knowles-Barley S., Lee D., Vázquez-Reina A., Kaynig V., Jones T., Roberts M., Morgan J., Tapia J., Seung H., Roncal W., Vogelstein J., Burns R., Sussman D., Priebe C., Pfister H., and Lichtman J., Saturated reconstruction of a volume of neocortex, Cell, 162 (2015), pp. 648–661. 26232230 10.1016/j.cell.2015.06.054

[21] Kazhdan M., Bolitho M., and Hoppe H., Poisson surface reconstruction, in Proceedings of the Fourth Eurographics Symposium on Geometry Processing, SGP ’06, Goslar, DEU, 2006, Eurographics Association, p. 61–70.

[22] Kazhdan M. and Hoppe H., Screened poisson surface reconstruction, ACM Trans. Graph., 32 (2013).

[23] Kingma D. P. and Ba J., Adam: A method for stochastic optimization, CoRR, abs/1412.6980 (2014).

[24] Kuwajima M., Mendenhall J. M., and Harris K. M., Large-volume reconstruction of brain tissue from high-resolution serial section images acquired by SEM-based scanning transmission electron microscopy, in Nanoimaging, Methods in molecular biology (Clifton, N.J.), Humana Press, Totowa, NJ, 2013, pp. 253–273. 10.1007/978-1-62703-137-0_15 PMC3716574 23086880

[25] LeCun Y., Bengio Y., and Hinton G., Deep learning, Nature, 521 (2015), pp. 436–44. 26017442 10.1038/nature14539

[26] Lee T., Kashyap R., and Chu C., Building skeleton models via 3-d medial surface axis thinning algorithms, CVGIP: Graphical Models and Image Processing, 56 (1994), pp. 462–478.

[27] Li H., Zhang Z., Li T., and Si X., A review on physics-informed data-driven remaining useful life prediction: Challenges and opportunities, Mechanical Systems and Signal Processing, 209 (2024), p. 111120.

[28] Lu L., Meng X., Mao Z., and Karniadakis G. E., Deepxde: A deep learning library for solving differential equations, SIAM Review, 63 (2021), pp. 208–228.

[29] Merino-Serrais P., Benavides-Piccione R., Blazquez-Llorca L., Kastanauskaite A., Rabano A., Avila J., and DeFelipe J., The influence of phospho-tau on dendritic spines of cortical pyramidal neurons in patients with alzheimer’s disease, Brain, 136 (2013), pp. 1913–1928. 23715095 10.1093/brain/awt088 PMC3673457

[30] Meyer M., Desbrun M., SchrÖder P., and Barr A. H., Discrete differential-geometry operators for triangulated 2-manifolds, in Visualization and Mathematics III, Hege H.-C. and Polthier K., eds., Berlin, Heidelberg, 2003, Springer Berlin Heidelberg, pp. 35–57.

[31] O’Neill B., Elementary Differential Geometry, Academic Press, 1997.

[32] Pchitskaya E., Vasiliev P., Smirnova D., Chukanov V., and Bezprozvanny I., SpineTool is an open-source software for analysis of morphology of dendritic spines, Sci. Rep., 13 (2023), p. 10561. 37386071 10.1038/s41598-023-37406-4 PMC10310755

[33] Raissi M., Perdikaris P., and Karniadakis G., Physics-informed neural networks: A deep learning framework for solving forward and inverse problems involving nonlinear partial differential equations, Journal of Computational Physics, 378 (2019), pp. 686–707.

[34] Rao C., Sun H., and Liu Y., Physics-informed deep learning for incompressible laminar flows, Theoretical and Applied Mechanics Letters, 10 (2020), pp. 207–212.

[35] Samaniego E., Anitescu C., Goswami S., Nguyen-Thanh V., Guo H., Hamdia K., Zhuang X., and Rabczuk T., An energy approach to the solution of partial differential equations in computational mechanics via machine learning: Concepts, implementation and applications, Computer Methods in Applied Mechanics and Engineering, 362 (2020), p. 112790.

[36] Smirnov M. S., Garrett T. R., and Yasuda R., An open-source tool for analysis and automatic identification of dendritic spines using machine learning, PLoS One, 13 (2018), p. e0199589. 29975722 10.1371/journal.pone.0199589 PMC6033424

[37] Spruston N., Pyramidal neurons: dendritic structure and synaptic integration, Nature Reviews Neuroscience, 9 (2008), pp. 206–221. 18270515 10.1038/nrn2286

[38] Su R., Sun C., Zhang C., and Pham T. D., A novel method for dendritic spines detection based on directional morphological filter and shortest path, Computerized Medical Imaging and Graphics, 38 (2014), pp. 793–802. 25155696 10.1016/j.compmedimag.2014.07.006

[39] Sullivan J. M., Curvatures of smooth and discrete surfaces, 2007.

[40] Tibshirani R., Regression shrinkage and selection via the lasso, Journal of the royal statistical society series b-methodological, 58 (1996), pp. 267–288.

[41] Ustinova A., Volkova E., Rakovskaya A., Smirnova D., Korovina O., and Pchitskaya E., Generate and analyze three-dimensional dendritic spine morphology datasets with SpineTool software, Curr. Protoc., 4 (2024), p. e70061. 39641661 10.1002/cpz1.70061

[42] van der Walt S., SchÖnberger J. L., Nunez-Iglesias J., Boulogne F., Warner J. D., Yager N., Gouillart E., Yu T., and scikit-image contributors, scikit-image: image processing in python, PeerJ, 2 (2014), p. e453. 25024921 10.7717/peerj.453 PMC4081273

[43] Vidaurre-Gallart I., Fernaud-Espinosa I., Cosmin-Toader N., Talavera-Martínez L., Martin-Abadal M., Benavides-Piccione R., Gonzalez-Cid Y., Pastor L., DeFelipe J., and García-Lorenzo M., A deep learning-based workflow for dendritic spine segmentation, Frontiers in Neuroanatomy, Volume 16 - 2022 (2022). 10.3389/fnana.2022.817903 PMC8967951 35370569

[44] Virtanen P., Gommers R., Oliphant T. E., Haberland M., Reddy T., Cournapeau D., Burovski E., Peterson P., Weckesser W., Bright J., van der Walt S. J., Brett M., Wilson J., Millman K. J., Mayorov N., Nelson A. R. J., Jones E., Kern R., Larson E., Carey C. J., Polat İ, Feng Y., Moore E. W., VanderPlas J., Laxalde D., Perktold J., Cimrman R., Henriksen I., Quintero E. A., Harris C. R., Archibald A. M., Ribeiro A. H., Pedregosa F., van Mulbregt P., and SciPy 1. 0 Contributors, SciPy 1.0: fundamental algorithms for scientific computing in Python, Nature Medicine, 17 (2020), pp. 261–272. 10.1038/s41592-019-0686-2 PMC7056644 32015543

[45] Vogel F. W., Alipek S., Eppler J.-B., Osuna-Vargas P., Triesch J., Bissen D., Acker-Palmer A., Rumpel S., and Kaschube M., Utilizing 2d-region-based CNNs for automatic dendritic spine detection in 3D live cell imaging, Scientific Reports, 13 (2023), p. 20497. 37993550 10.1038/s41598-023-47070-3 PMC10665560

[46] Wu C.-H., Fai T. G., Atzberger P. J., and Peskin C. S., Simulation of osmotic swelling by the stochastic immersed boundary method, SIAM Journal on Scientific Computing, 37 (2015), pp. B660–B688.

[47] Xiao X., Djurisic M., Hoogi A., Sapp R. W., Shatz C. J., and Rubin D. L., Automated dendritic spine detection using convolutional neural networks on maximum intensity projected microscopic volumes, Journal of Neuroscience Methods, 309 (2018), pp. 25–34. 30130608 10.1016/j.jneumeth.2018.08.019 PMC6402488

[48] Yu W. and Lu B., Synapses and dendritic spines as pathogenic targets in alzheimer’s disease, Neural plasticity, 2012 (2012), p. 247150. 22474602 10.1155/2012/247150 PMC3306944

[49] Yuksel C., Sample elimination for generating poisson disk sample sets, Comput. Graph. Forum, 34 (2015), p. 25–32.

[50] Yuste R., Dendritic spines and distributed circuits, Neuron, 71 (2011), pp. 772–781. 21903072 10.1016/j.neuron.2011.07.024 PMC4071954

[51] Zhang T. Y. and Suen C. Y., A fast parallel algorithm for thinning digital patterns, Commun. ACM, 27 (1984), pp. 236–239.

[52] Zhou Q.-Y., Park J., and Koltun V., Open3D: A modern library for 3D data processing, arXiv:1801.09847, (2018).

